# Copaiba Oil-Loaded Polymeric Nanocapsules: Production and In Vitro Biosafety Evaluation on Lung Cells as a Pre-Formulation Step to Produce Phytotherapeutic Medicine

**DOI:** 10.3390/pharmaceutics15010161

**Published:** 2023-01-03

**Authors:** Victor M. Rodrigues, Wógenes N. Oliveira, Daniel T. Pereira, Éverton N. Alencar, Dayanne L. Porto, Cícero F. S. Aragão, Susana M. G. Moreira, Hugo A. O. Rocha, Lucas Amaral-Machado, Eryvaldo S. T. Egito

**Affiliations:** 1 Graduate Program in Health Sciences, Federal University of Rio Grande do Norte (UFRN), Natal 59012-570, Brazil; 2 Graduate Program in Pharmaceutical Nanotechnology, Federal University of Rio Grande do Norte (UFRN), Natal 59012-570, Brazil; 3 Pharmacy Department, Federal University of Rio Grande do Norte (UFRN), Natal 59012-570, Brazil; 4 Graduate Program in Pharmaceutical Sciences, Federal University of Rio Grande do Norte (UFRN), Natal 59012-570, Brazil; 5 Department of Cellular and Molecular Biology, Biosciences Center, Federal University of Rio Grande do Norte (UFRN), Natal 59078-900, Brazil; 6 Laboratory of Natural Polymers Biotechnology, Federal University of Rio Grande do Norte (UFRN), Natal 59078-900, Brazil

**Keywords:** copaiba oil, polymeric nanocapsules, cytotoxicity, genotoxicity

## Abstract

Copaiba oil has been largely used due to its therapeutic properties. Nanocapsules were revealed to be a great nanosystem to carry natural oils due to their ability to improve the bioaccessibility and the bioavailability of lipophilic compounds. The aim of this study was to produce and characterize copaiba oil nanocapsules (CopNc) and to evaluate their hemocompatibility, cytotoxicity, and genotoxicity. Copaiba oil was chemically characterized by GC-MS and FTIR. CopNc was produced using the nanoprecipitation method. The physicochemical stability, toxicity, and biocompatibility of the systems, in vitro, were then evaluated. Β-bisabolene, cis-α-bergamotene, caryophyllene, and caryophyllene oxide were identified as the major copaiba oil components. CopNc showed a particle size of 215 ± 10 nm, a polydispersity index of 0.15 ± 0.01, and a zeta potential of −18 ± 1. These parameters remained unchanged over 30 days at 25 ± 2 °C. The encapsulation efficiency of CopNc was 54 ± 2%. CopNc neither induced hemolysis in erythrocytes, nor cytotoxic and genotoxic in lung cells at the range of concentrations from 50 to 200 μg·mL^−1^. In conclusion, CopNc showed suitable stability and physicochemical properties. Moreover, this formulation presented a remarkable safety profile on lung cells. These results may pave the way to further use CopNc for the development of phytotherapeutic medicine intended for pulmonary delivery of copaiba oil.

## 1. Introduction

Copaiba oil (Cop) is a natural oil extracted from plants of the genus *Copaifera*, which can be found in South America. Different species are present in this continent, such as *Copaifera officinalis*, which was used in this study [1]. Cop is commonly used as a folk medicine for the treatment of pulmonary inflammations, such as bronchitis and asthma [1,2]. In addition, it presents other biological properties such as analgesic, antinociceptive, antimicrobial, and gastroprotective. These different biological activities are attributed to the presence of Cop’s chemical composition of several metabolites (traditionally named secondary metabolites), mainly terpenoid compounds, such as sesquiterpenes (β-caryophyllene, β-bisabolene, and α-humulene) and diterpenes (kaurenoic acid, kaurenol, and copalic acid) [3,4,5,6].

Despite these remarkable Cop therapeutic properties, its in natura use is unsuitable for topical or oral applications due to the undesirable organoleptic characteristics and physicochemical and biopharmaceutical properties [7]. In this context, nanostructured systems, such as polymeric nanocapsules, stand out as a promising alternative for the obtention of formulations containing natural oils, once they can mitigate the organoleptic properties of these compounds, such as the taste and smell. In addition, nanostructured systems can also encapsulate and modify the bioavailability of natural oils by different mechanisms, including (i) protecting the oils from degradation; (ii) enhancing cellular uptake and apparent solubility on body fluids; and (iii) promoting an effective drug release on different tissues, such as the lungs [8,9].

Notwithstanding Cop’s suitable therapeutic characteristics and possible delivery by polymeric nanocapsules, only a few studies demonstrate the biocompatibility/biosafety of the oil when loaded into nanostructured systems. Indeed, the biocompatibility of these systems is a primordial requirement prior to their use. Nanostructured systems can directly interact with cells and the extracellular environment, which can trigger different biological effects, such as changes in cell metabolism or undesirable effects on human tissues [10]. Therefore, once natural oils have a complex chemical matrix, their loading on nanostructured systems could change their pharmacodynamic and safety profile. Such a feature is possible due to the increase in the apparent oil solubility, enhancing its bioavailability, which increases the exposure of oil components to cellular structures more efficiently than traditional medicines, resulting in increased pharmacological effects [10,11]. Then, considering the aforementioned conditions, the biocompatibility of natural oils-loaded nanostructured systems should be assessed.

In light of this, health surveillance agencies and international organizations, such as the Organization for Economic Co-operation and Development (OECD), suggest tests that can be used and adapted to assess the biosafety of any medical device, drug, or food, such as cytotoxicity and genotoxicity assays [12,13]. These are important aspects to be evaluated, since different plant compounds or natural oils may show cytotoxic effects or cause DNA damages that could lead to the development of mutations, which ultimately could induce cell proliferation malfunctions [14]. Then, the biosafety evaluation stands out as a primordial step in the development of nanosystems intended for natural oil delivery.

Therefore, the aim of this study was to produce and characterize copaiba oil nanocapsules (CopNc) with *Copaifera officinalis* oil and to evaluate their hemocompatibility, cytotoxicity, and genotoxicity using in vitro tests with human erythrocytes and alveolar epithelial lung A549 cells, respectively.

## 2. Materials and Methods

### 2.1. Materials

MTT (3-(4,5-Dimethylthiazol-2-yl)-2,5-Diphenyltetrazolium Bromide), polycaprolactone (PCL; molecular weight: 80,000), Span^®^ 60 (sorbitan monooleate 60), Tween^®^ 80 (polysorbate 80), cytochalasin B (Cyt B), mitomycin C, anhydrous pyridine, β-caryophyllene (98%), and N,O-bis(trimethylsilyl)trifluoroacetamide (BSTFA) were purchased from Sigma-Aldrich, Inc. (St. Louis, MO, USA). Dulbecco’s modified eagle’s medium (DMEM) and fetal bovine serum (FBS) were from ThermoFischer-Gibco (Gaithersbug, MD, USA). Analytical-grade acetone was purchased from Vetec (Rio de Janeiro, RJ, Brazil). Acetonitrile was from Dinamica^®^ (São Paulo, SP, Brazil). N-heptane 99% (Neon, Suzano, SP, Brazil) was donated by the Agricultural School of Jundiaí (EAJ) from the Federal University of Rio Grande do Norte (UFRN) (Macaíba, RN, Brazil). A549 cells (ATCC^®^, Rockville, MD, USA) were gently donated by the Laboratory of Molecular Biology and Genomics (UFRN) (Natal, RN, Brazil). *Copaifera officinalis* oil was purchased from the Laboratório São Lucas (Belém, PA, Brazil) and its use in this study was approved after registration in the Sistema Nacional de Gestão de Patrimônio Genético e do Conhecimento Tradicional Acumulado (SisGen) (number A5F3DA7).

### 2.2. Copaiba Oil Chemical Characterization

#### 2.2.1. Gas Chromatography—Mass Spectroscopy (GC-MS)

The chemical composition of Cop was determined using a gas chromatographic coupled to a mass spectrometer (GC-MS) (Agilent, Santa Clara, CA, USA). Copaiba oil was directly diluted with n-heptane to achieve a 1 mg·mL^−1^ concentration, filtered through a 0.2 µm syringe filter, placed in a vial, and 1 µL was injected into an HPMS-5MS silica capillary column (part number 19091S-433) (Agilent, Santa Clara, CA, USA). This method was adapted from a previously validated study by our research group [15]. In addition, to analyze the presence of the diterpenes in the oil composition, Cop was derivatized by silylation using N,O-bis(trimethylsilyl)trifluoroacetamide (BSTFA) reagent. Briefly, Cop (500 μL) was added into a vial containing 600 μL of anhydrous pyridine and homogenized by magnetic stirring (IKA C-MAG HS 7, Staufen, Germany) at 200 rpm and 60 °C. Moreover, the oil was derivatized using 400 μL of the BSTFA and the mixture was kept under magnetic stirring for 10 min. Thus, the sample was diluted 1:10 _(*v*/*v*)_ in anhydrous pyridine and 2 μL of the mixture was injected into an HPMS-5MS silica capillary column. For both analyses, the injector and the column were set at 250 °C and 60 °C, respectively. A heating ramp of 3 °C·min^−1^ to 240 °C, followed by a second heating at 5 °C·min^−1^ to 250 °C, remained isothermal for 5 min after the final temperature was reached. The carrier gas helium was used at a flow rate of 1 mL·min^−1^. The split ratio was 1:25, with the electron ionization system set at 70 eV. The mass fragmentation of Cop components was identified by comparison to a mass spectral electronic library (National Institute of Standards and Technology—NIST) and an analysis of previous data published by the literature [15].

#### 2.2.2. Fourier-Transform Infrared Spectroscopy (FTIR)

Cop’s chemical characterization was also assessed by infrared spectroscopy, using a Prestige-21 FTIR spectrophotometer (Shimadzu Corporation, Kyoto, Japan) at 25 ± 2 °C. The sample was placed in a ZnSe crystal and the spectra were recorded from 4000 to 700 cm^−1^, with a resolution of 4 cm^−1^, and there were 20 scans accumulated.

### 2.3. Copaiba Oil Nanocapsules Production

CopNc were produced using the nanoprecipitation methods first described by Fessi et al. 1989 [16]. Thereafter, two phases (aqueous and organic) were prepared separately. The aqueous phase was composed of Tween^®^ 80 (77 mg) and purified water (53 mL). The organic phase was produced with acetone (27 mL), Cop (160 mg), PCL (100 mg), and Span^®^ 60 (38 mg). Thus, the two phases were mixed separately under moderate magnetic stirring (IKA^®^ RO 15, Staufen, BW, Germany) at 40 °C until complete dissolution. Posteriorly, the organic phase was continuously poured into the aqueous phase, followed by homogenization for at least 10 min. Then, the organic solvent was eliminated, and nanoparticles were concentrated at 40 °C by rotary evaporation (IKA^®^ RV 10 basic, Staufen, BW, Germany). Finally, the remaining volume was adjusted to 10 mL using purified water.

### 2.4. Physicochemical Characterization and Stability Evaluation of the Nanocapsules

Beforehand, β-caryophyllene, one of the major components of Cop was used as the standard for quantification of Cop from CopNc. Cops’ inherent amount of β-caryophyllene was previously determined. Then, a known amount of this compound was further added to the oil to improve quantification based on this analyte. The aforementioned chromatography conditions were used for quantification. A β-caryophyllene standard curve (a concentration range of 40 to 160 µg·mL^−1^) in n-heptane was constructed, which was adapted from Xavier-Jr. et al. (2017) [15]. Afterward, the CopNc oil fraction was analyzed posterior to a solvent–solvent extraction, as follows. First, acetonitrile was used to dissolve the polymeric particle; then, water was added to reduce the non-polar solubility in the solvent mixture; and finally, the oil fraction was extracted with n-heptane under centrifugation at 9800 RCF using Gusto^®^ High-Speed mini centrifuge (Heathrow Scientific, Vernon Hills, IL, USA). The non-polar solvent layer (n-heptane) was used in the GC-MS for Cop quantification using the standard curve equation (R^2^: 0.991). Indirect encapsulation efficiency (based on β-caryophyllene amounts) was determined according to Equation (1):

(1) $$Β\mathrm{-Caryophyllene}\;\left[\;\;\right]=\frac{\mathrm{Peak}\;\mathrm{area}\;+\;165,541}{51,503}$$

where peak area is the integrated area of the β-caryophyllene peak on GC chromatograms, 165,541 is the intersection, and 51,503 is the inclination value of the standard curve.

Furthermore, encapsulation efficiency (EE%) was calculated based on the above-determined concentration and dilution parameters, using Equation (2):

(2) $$\mathrm{EE}\%=\frac{\mathrm{BC}}{\mathrm{NBC}\cdot \mathrm{EC}}\times 100$$

where BC is the β-caryophyllene concentration, NBC is the nominal concentration of β-caryophyllene (Copaiba oil + Internal standard), and EC is the extraction coefficient, which, in this case, is 0.6750.

Additionally, to qualitatively investigate the loading of Cop into the system, individual FTIR analyses of unloaded-CopNc, CopNc, Span^®^ 60, Tween^®^ 80, and PCL were performed according to the parameters mentioned in Section 2.2.2.

Furthermore, CopNc physicochemical characterization and stability were assessed through particle size distribution and zeta potential. The particle size distribution was determined by dynamic light scattering (DLS). The samples were diluted at 1:100 _(*v*/*v*)_ in purified water before analysis using a ZetaSize NanoZS instrument (Malvern Instruments, Malvern, UK), pre-calibrated at 25 °C, and an angle fixed at 173°. Additionally, the nanocapsules were diluted at 1:500 _(*v*/*v*)_ in a sodium chloride solution (0.1 mM) to preserve the ionic strength and had their electrophoretic mobility measured at 25 °C to assess the zeta potential. All measurements were performed in triplicate. Finally, CopNc was stored at room temperature (25 ± 2 °C) and a humidity rate of 75%. It was continuously monitored by a thermo-hygrometer and then re-evaluated according to the mentioned parameters for 30 days.

### 2.5. Biocompatibility Assessment of Cop and CopNc

#### 2.5.1. Hemolytic Potential Evaluation

**Scheme 1:**
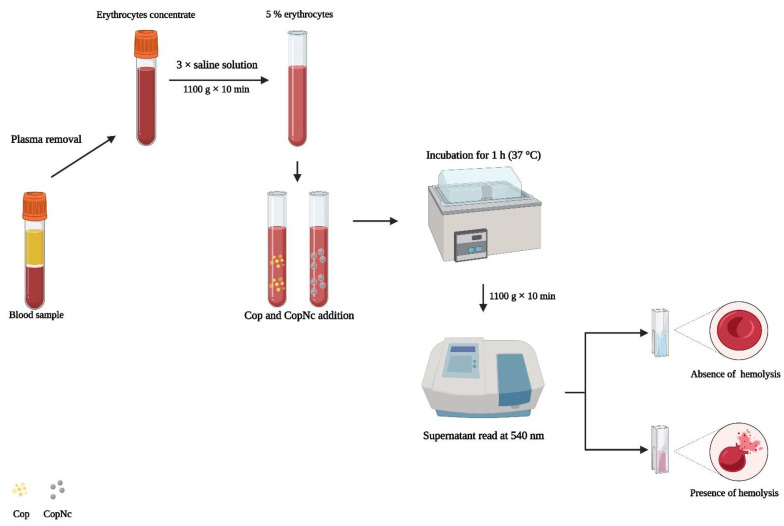
Representation of the experimental protocol for determining the hemolytic potential evaluation of Cop and CopNc on human erythrocytes.

To evaluate the hemolytic potential of Cop and CopNc, the method proposed by Oliveira et al. (2018) was performed [17]. Therefore, an O^+^ blood sample was collected from a healthy human donor, after ethical committee approval (number 3.690.763—Research Ethical Committee of the Federal University of Rio Grande do Norte) and patient consent. Posteriorly, the blood was centrifuged at 1100 g for 10 min, followed by plasma removal. Then, the erythrocytes concentrate was washed three times using a saline solution at 0.9% and the volume was adjusted to a suspension containing 5% _(*v*/*v*)_ of erythrocytes (6 × 10^7^ cells). Thereafter, 1.5 mL of the erythrocytes suspension was incubated with 1.5 mL of Cop and CopNc dispersions at concentrations of 50, 100, 150, 200, 250, 300, and 500 μg.mL^−1^ for 1 h at 37 °C (Scheme 1). To reach these concentrations, Cop was diluted in a saline solution with 1% DMSO and CopNc were diluted in a saline solution considering the theoretical amount of Cop in the system. After incubation, the samples were centrifuged at 1100 g for 10 min and the released hemoglobin on the supernatant was evaluated in a spectrophotometer set at 540 nm. Triton-X100 at 1% and a saline solution were used as positive and negative controls, respectively. Analyses were performed in triplicate and results were calculated according to Oliveira et al., 2018 [17].

#### 2.5.2. Evaluation of MTT Reduction by Mitochondria Enzymes

The alveolar epithelial cell line A549 was chosen for in vitro assessment of the biocompatibility of Cop and CopNc. Cells were maintained in a DMEM basal culture medium (Dubecco’s Essential Medium Eagle, GIBCO^®^, Thermo Scientific, Waltham, MA, USA), supplemented with 10% fetal bovine serum (FBS), 1% antibiotics (10,000 U·mL^−1^ penicillin G and 25 μg·mL^−1^ streptomycin), and 1% glutamine. A549 cells were incubated and cultured at 37 °C and a 5% CO_2_ atmosphere.

**Scheme 2:**
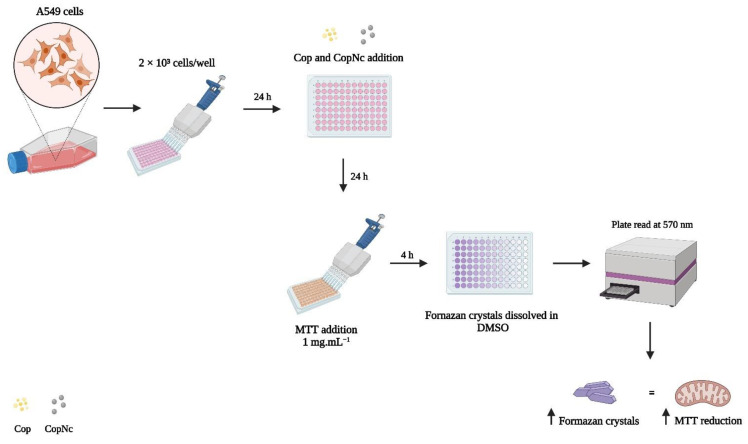
Representation of the experimental protocol for the cytotoxicity assay based on the MTT reduction by mitochondria enzymes of Cop and CopNc on A549 cells.

The MTT assay was performed to assess the cytotoxicity of Cop and CopNc against A549 cells (Scheme 2). The cells were placed in 96-well plates (2 × 10^3^ cells/well) and incubated for 24 h in a basal culture medium. A DMSO aqueous solution (1% _(*v*/*v*)_) of Cop at 2 mg·mL^−1^ and an aqueous dispersion of CopNc, based on the theoretical amount of Cop in the same concentration, were prepared as stock solutions. Subsequently, samples were diluted in DMEM to reach final Cop and CopNc concentrations of 50, 100, 150, 200, 250, 300, and 500 μg·mL^−1^. Diluted samples were then added to the cells, followed by incubation for 24 h. After the incubation period, the medium containing the samples was removed and 100 μL of phosphate-buffered saline (PBS) containing the MTT reagent (1 mg·mL^−1^) was added, followed by 4 h of incubation. The formazan crystals formed by the MTT salt reduction were diluted in 200 μL of DMSO. Finally, the absorbance was evaluated using an Elisa Multiskan Ascent Microplate Reader (Thermo Labsystems, Franklin, MA, USA) set at 570 nm. Untreated cells were used as a negative control and the MTT reduction (%) by mitochondria enzymes were determined by the relative absorbance between the untreated cells and the samples. This assay was performed in triplicate.

#### 2.5.3. Cytokinesis Block Micronucleus Assay (CBMN)

**Scheme 3:**
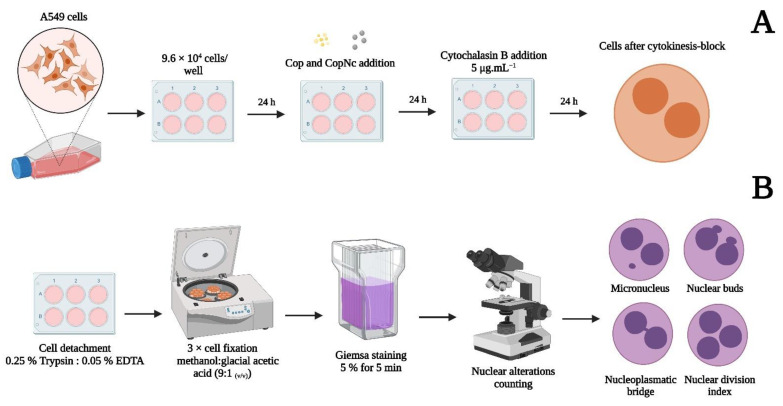
Representation of the experimental protocol for genotoxicity evaluation of Cop and CopNc on A549 cells using the CBMN assay. (**A**) Morphology of binucleated cells before detachment and staining; (**B**) Morphology of fixed cells after Giemsa staining.

To evaluate the genotoxic potential of Cop and CopNc, the CBMN assay was adapted from the guidelines for the in vitro mammalian cell micronucleus test and developed according to the Fenech protocol with some modifications (Scheme 3) [13,18]. Thus, A549 cells were seeded in a 6-well plate (9.6 × 10^4^ cells/well) and incubated for 24 h prior to treatment with 200 μg·mL^−1^ of Cop and CopNc, prepared as previously described. Then, the medium containing the samples was removed and cytochalasin B (5 μg·mL^−1^) was added and incubated for an additional 24 h to stop cytokinesis. Thereafter, cells were washed with PBS, detached using 0.25% trypsin and 0.05% EDTA, suspended in a culture medium, and finally fixed by three incubations with methanol:glacial acetic acid (9:1 _(*v*/*v*)_). Subsequently, 80 μL of each cell suspension was placed on clean air-dried slides and stained with an aqueous solution of 5% Giemsa for 5 min. Finally, the slides were analyzed by optical microscope following the criteria for counting and characterizing micronuclei (MN), nuclear buds (NBUDs), nucleoplasmatic bridges (NPBs), and mononuclear and multi-nuclear cells, as proposed by Fenech [18]. The nuclear division index (NDI) was determined for each condition. Cells treated with mitomycin C (1 μg·mL^−1^ simultaneously added with cytochalasin-B) for 24 h were used as the positive control and untreated cells were used as the negative control. A total of 3000 binuclear cells per treatment were analyzed in three independent experiments and results were expressed by alterations occurrence per 1000 binucleated cells.

### 2.6. Statistical Analyses

Results were expressed as mean ± standard deviation. Statistical analyses were carried out by one-way analysis of variance (ANOVA) with Dunnet’s post-test for the hemolysis and the MTT assay. In addition, a two-way analysis of variance (ANOVA) with Dunnet’s post-test was carried out for the CBMN assay using GraphPad Prism version 8.02 (San Diego, CA, USA). Differences in the mean values with * *p* < 0.05 were considered statistically significant.

## 3. Results and Discussion

### 3.1. Copaiba Oil Chemical Characterization

The chemical characterization of Cop was performed to identify the major compounds presented in its lipidic matrix. Indeed, several techniques are available to analyze natural oils’ chemical composition; however, GC-MS stands out due to its remarkable performance in the separation, identification, and quantification of individual compounds present in natural oils [19]. In addition, Cop is usually composed of two main fractions, a volatile and a non-volatile one, and both fractions can be identified using GC-MS, which makes this technique the best choice for Cop characterization [15].

Based on the GC-MS results (Table 1), it is possible to observe the presence of many terpenoid molecules, which are commonly found in natural oils derived from plants of the genus Copaifera [20]. Among those molecules, the major compounds identified were β-bisabolene (50.2%) and cis-α-bergamotene (23.1%) in Cop and β-bisabolene (36.9%) and α-bergamotene (22.3%) in the derivated oil (SiCop); both sesquiterpenes are usually described for C. officinalis natural oil. In addition, the derivatization of the oil was performed to highlight the presence of diterpenes, such as Kaur-16-en-18-al, (4α)-(3.3%), and fatty acids, such as linoleic acid (4.8%), since they are also commonly found in natural oils [21]. The results also showed the presence of β-caryophyllene (5.1%), which along with β-bisabolene and α-bergamotene, are frequently used as chemical analytes for Copaifera-obtained oils.

Similar to our results, Cascon et al. (2000), who analyzed the chemical composition of oils from C. multijuga, C. guianensis, and C. duckei using GC-MS, also found the presence of sesquiterpenes in these three oils. Nonetheless, the authors reported that C. multijuga and C. guianensis displayed an amount of β-caryophyllene superior to 40%, and β-bisabolene and α-bergamotene amounts lower than 7% of the oils’ total compositions. In addition, for C. duckei, the presence of β-caryophyllene, β-bisabolene, and α-bergamotene was around 5%, 10%, and 8%, respectively [22]. These results, compared to the herein obtained, differ in the quantification of major compounds and allow us to reinforce the heterogeneity of specialized metabolites on the composition of plants of the genus Copaifera, which can be related to climatic conditions and natural genetic variations. Consequently, these variations in the oil composition can influence its biological activity and toxicity [23]. Therefore, the identification of natural oils’ chemical composition has to be a primordial step previous to biological studies. Finally, it is important to highlight that, even with these observed variations, the main bioactive molecules of the Copaifera genus are presented, assuring their identity, as they have been previously studied by our research group. In addition, the biological effect of these identified compounds suggests the use of this oil for the treatment of inflammatory disorders, as described by Caputo et al. (2020) in a model of pulmonary inflammation and by Ghizoni et al. (2017) in a model of arthritis [24,25,26].

**Figure 1:**
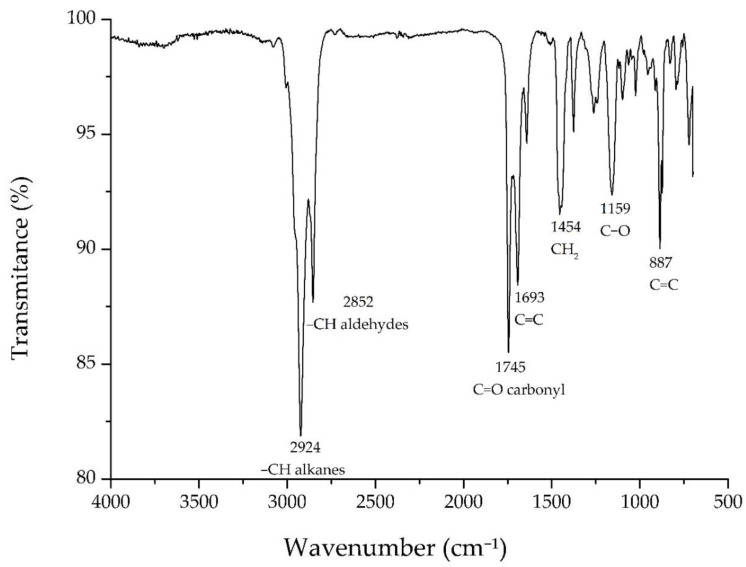
A Fourier-transform infrared spectroscopy (FTIR) evaluation of copaiba oil (Cop).

Furthermore, the presence of chemical groups related to the compounds, identified by GC-MS, was confirmed by FTIR (Figure 1). Two intense peaks were observed at 2924 and 2852 cm^−1^, attributed to the symmetric stretching of the –CH bonds in alkanes and aldehydes, respectively. In addition, the peak at 1745 cm^−1^ suggests carbonyl stretching and the peak at 1693 cm^−1^ suggests axial deformation of C=C bonds. Moreover, the peaks at 1454, 1159, and 887 cm^−1^ indicate the bending vibrations of aliphatic CH_2_, C–O bond vibrations and C=C bending, respectively. These results reinforce the presence of sesquiterpenes, such as β-bisabolene and β-caryophyllene, already identified in Cop’s chemical composition by GC-MS (Table 1), which is in agreement with previous reports in the literature [20,23,27,28].

Overall, Cop chemical characterization highlighted the presence of terpenic compounds commonly identified in several species of the Copaifera genus [20], which are frequently found in Cop extracted from plants of north, northeast, and midwest areas of Brazil [29]. These molecules are also responsible for the anti-inflammatory and antioxidant activities described for Cop in the respiratory system [30]. Then, both GC-MS and FTIR analyses confirmed a suitable Cop chemical composition, which reinforces its use in the production of pharmaceutical systems intended for pharmacological use. Therefore, Cop-loaded polymeric PCL nanocapsules were produced and characterized.

### 3.2. Copaiba Oil Nanocapsules—Production and Physicochemical Characterization

CopNc was produced by the nanoprecipitation method proposed by Fessi et al., 1989 [16]. This well-established and reproducible technique allows the encapsulation of natural oils into the core of polymeric nanocapsules and enables their administration in different human tissues [31]. In addition, the CopNc system was produced using PCL, a polymer approved by the Food Drug Administration (FDA) to develop therapeutic products for internal use due to its low toxicity and suitable biocompatibility [32]. After the production of CopNc, the oil encapsulation efficiency was determined, and FTIR analyses of unloaded-CopNc (without Cop), CopNc, Span 60^®^, Tween 80^®,^ and PCL were conducted. Moreover, the system was characterized by particle size distribution and zeta potential, which were evaluated over 30 days at room temperature (25 ± 2 °C). 

Encapsulation efficiency (EE%) of CopNc was obtained by using the β-caryophyllene standard curve for indirect oil quantification in the samples. EE% represented 54 ± 2% of the total oil used for the production. Other major compounds were also qualitatively identified in the sample (i.e., copalic acid), corroborating Cop’s loading in the nanostructured system. Xavier-Jr. et al. (2018) developed copaiba oil-loaded polylactic acid (PLA) nanocapsules and obtained an encapsulation efficiency of approximately 70%, which indicates that the oil was immediately diffused into the internal phase of nanocapsules, in which it was entrapped by the nanocapsules’ polymeric envelope [33]. Moreover, the presence of the quantified β-caryophyllene, along with the other qualitatively identified compounds inside CopNc, highlights the maintenance of the oil composition after the nanoencapsulation process, which is important since these molecules altogether are responsible for the biological effects of Cop, as previously mentioned [24,33]. 

**Figure 2:**
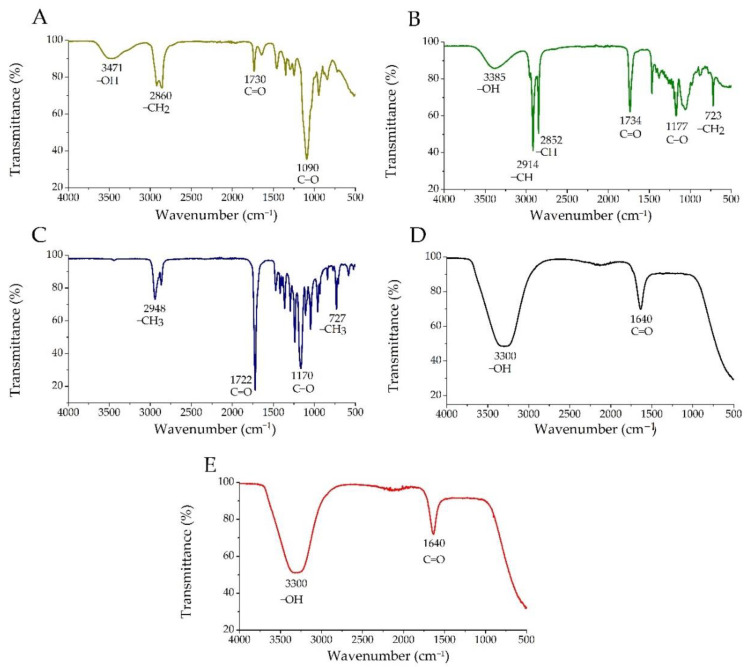
A Fourier-transform infrared spectroscopy (FTIR) evaluation of CopNc and each separate component of the system. (**A**): A Tween 80^®^ spectrum; (**B**): a Span 60^®^ spectrum; (**C**): a PCL spectrum; (**D**): an unloaded-CopNc spectrum; and (**E**): a CopNc spectrum.

Moreover, FTIR analyses of unloaded-CopNc, CopNc, and all formulation components were performed in order to investigate Cop encapsulation by the nanocapsules (Figure 2). The Tween 80^®^ spectrum (Figure 2A) showed characteristic peaks at 3471 (–OH stretching), 2860 (–CH_2_ stretching), 1730 (C=O stretching), and 1090 cm^−1^ (C–O stretching). Moreover, the Span 60^®^ spectrum (Figure 2B) also showed characteristic peaks at 3385 (–OH stretching), 2914, and 2852 (–CH stretching, asymmetric and symmetric, respectively), as well as 1734 (C=O stretching), 1177 (C–O stretching), and 723 cm^−1^ (–CH_2_ aliphatic stretching). The PCL spectrum (Figure 2C) showed typical bands of the polymer at 2948 (–CH_3_ stretching), 1722 (C=O stretching), 1170 (C–O asymmetric stretching), and 723 cm^−1^ (–CH_3_ stretching). These results are in agreement with previously reported data and predict the molecular integrity of all formulation components [34,35]. 

The FTIR spectra of unloaded-CopNc (Figure 2D) and CopNc (Figure 2E) were also obtained. Both results showed two main peaks at 3300 and 1640 cm^−1^, which could be attributed to –OH stretching of the surfactants and C=O stretching of PCL polymer, respectively. In addition, it was possible to notice that both systems showed very similar profiles, which qualitatively suggests the ability of CopNc to properly encapsulate Cop, due to the suppression of Cop peaks (Figure 1) in the analyses of the loaded system (Figure 2). In fact, Camargo et al. (2021), who developed tacrolimus-loaded PCL nanocapsules, also showed that the unloaded system had a similar FTIR profile compared to the tacrolimus-loaded system, which indicated drug encapsulation [36]. These results highlighted the ability of PCL nanocapsules to encapsulate different molecules and complex components, such as Cop [9]. Moreover, CopNc was subsequently produced and physicochemically characterized according to its particle size distribution and zeta potential.

After production (day 1), the CopNc system showed a liquid aspect with a slightly white opalescent color. In addition, it was observed with a particle size of 215 ± 10 nm and a PdI of 0.15 ± 0.01 (Table 2). These values remained similar throughout 30 days of analysis (*p* > 0.05), confirming the successful production and suitable physicochemical stability of CopNc under a preformulation assessment [37]. Indeed, the average particle size of PCL nanocapsules produced by nanoprecipitation is commonly around 200 nm. A similar result was observed in the work of Camargo et al. (2021), who obtained nanocapsules with a particle size of approximately 220 nm using the same production method [36]. Moreover, the nanocapsules’ particle size is strongly affected by the chosen polymer and method of production, as can be observed in the work of Łukasiewicz et al. (2021), who produced nanocapsules using pegylated and non-pegylated PCL through a nanoemulsion templating method and obtained a particle size of approximately 90 nm [38], a value distinct from the obtained by the nanoprecipitation method.

It is important to highlight the importance of particle size evaluation on nanocapsule development intended for pulmonary delivery, since this parameter affects the pharmacokinetic profile of nanocapsules, such as their residence time in the bloodstream, their toxicity, and their stability in organic fluids, such as lung compartments [31,39]. Indeed, the deposition of the particles over airways is a size-dependent mechanism, which is highlighted in deposition and clearance studies in the respiratory tract [40]. Furthermore, particles are mainly deposited onto the lung epithelium due to the combined effects of diffusion, sedimentation, and impaction. In regard to nanoparticles, diffusion is the main deposition mechanism and it significantly depends on particle size, lung morphology, and respiratory parameters. [40,41]. Significant amounts of nanoparticles are deposited in the alveoli as well as in the extrathoracic airways [42]. In addition, nanoparticles with approximately 200 nm are generally deposited in the lung lining fluid until dissolution, which allows them to escape from both phagocytic and mucociliary clearance mechanisms. Nonetheless, their drug release is severely limited because of their low inertia, which causes them to be predominantly exhaled from the lungs after inspiration [43]. Given this issue, some researchers frequently report that trojan microparticles, characterized by geometric sizes larger than 3 μm, when obtained from polymeric nanocapsules, are better suitable for the pulmonary route since they show desirable aerosolization efficiency [43,44]. Accordingly, our work presents itself as a “preformulation step”, once the next phases of the study are conducted by our research group to obtain a promising solid phytotherapeutic medicine composed of trojan microparticles.

Subsequently, the zeta potential of CopNc was also evaluated, once this parameter was related to the electrostatic stability of the developed nanocapsules. The obtained result of −18 ± 1 mV indicate a suitable electrostatic stability of CopNc. Indeed, zeta potential values lower than −10 mV allow suitable colloidal stability due to the electrostatic barrier between particles, which could prevent the occurrence of attraction forces and allow nanoparticles to remain dispersed [45]. Similar zeta potential values for PCL nanoparticles were found by both Khayata et al. (2012) and Byun et al. (2011), who produced vitamin E-loaded nanocapsules and obtained values of approximately −15 mV, which supports our results [45,46]. 

Therefore, CopNc showed not only suitable physicochemical stability over 30 days, but also promising physicochemical properties that could be advantageous to further produce a system for pulmonary delivery and to perform pharmacological studies. Nonetheless, previous to the evaluation of the pharmacological effects of CopNc, it is essential to ensure its biosafety on lung cells. 

### 3.3. Biocompatibility Assessment of Cop and CopNc

The biocompatibility assessment of Cop and CopNc was performed to evaluate the ability of both the oil and the nanocapsules to maintain homeostasis and normal features of lung cells in vitro, aiming a prospective pulmonary administration [40]. In this context, the hemolytic potential and the cytotoxic and genotoxic effects of Cop and CopNc were evaluated.

Indeed, the respiratory tract presents a large internal surface area and a very thin air–blood tissue barrier, which are essential for optimal diffusion-driven gas exchange between air and blood [41]. In this context, inhaled nanoparticles may also enter the respiratory tract and come into contact with blood cells [47]. Therefore, the hemocompatibility of Cop and CopNc was evaluated by the hemolytic potential assay, which is considered to be a simple and reliable method for estimating the blood compatibility of materials [48].

**Figure 3:**
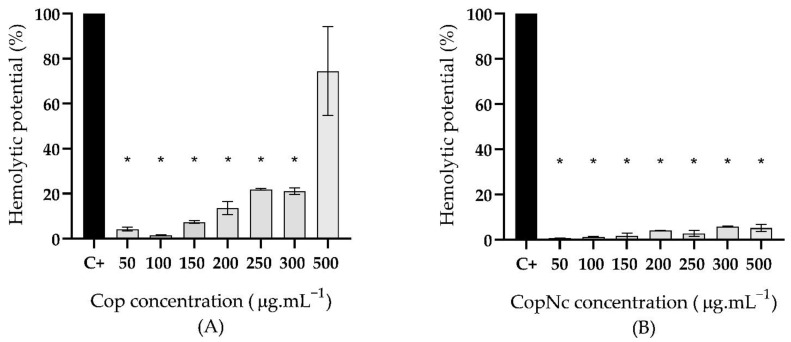
The hemolytic potential of free copaiba oil (Cop) (**A**) and copaiba oil-loaded nanocapsules (CopNc) (**B**) at concentrations from 50 to 500 μg·mL^−1^ based on the theoretical amount of Cop. C+: positive control; *: *p* < 0.05.

The hemolytic potential of Cop and CopNc was evaluated in a range of concentrations from 50 to 500 μg·mL^−1^ (Figure 3). Cop (Figure 3A) showed a dose-dependent hemolytic potential, wherein the hemolytic effect was lower than 15% at concentrations of 50, 100, 150, and 200 μg·mL^−1^ compared to the positive control (*p* < 0.05), suggesting a suitable hemocompatibility [17]. On the other hand, at 250 μg·mL^−1^ the hemolytic effect was higher than 15%, reaching 70 ± 19% of hemolysis at 500 μg·mL^−1^, showing a considerable hemolytic effect. This phenomenon can be attributed to the presence of terpenoids in Cop’s chemical composition since these compounds have been associated with the membrane disruption of human erythrocytes at high concentrations [49]. Izumi et al. (2012) demonstrated a hemolytic dose-dependent effect of terpenes from Cop, which showed 50% of hemolysis at concentrations above 400 μM. Thus, the hemolytic potential of Cop at concentrations above 200 μg.mL^−1^ could be also related to its terpenoid content [49]. In addition, the hemolytic potential effect observed at concentrations higher than 250 μg.mL^−1^ cannot be related to the DMSO stock solution at 1% used to prepare Cop samples. In fact, it is important to note that all used solvents to disperse or solubilize the samples were previously tested by our research group and their safety in biological assays was attested [9,17].

On the other hand, the results from CopNc (Figure 3B) showed a hemolytic potential lower than 15% at all tested concentrations. This result suggests an apparent protective effect of CopNc on blood cells, which could be related to the ability of the system to encapsulate the oil and delay its release. Consequently, the contact between the oil and the erythrocytes was decreased once the system could modulate the release of the oil and prevent the occurrence of hemolysis [39]. In fact, Lima et al. (2021), who developed microparticles with different natural oils, showed that copaiba oil microparticles were hemolytic only at a concentration of 1000 μg·mL^−1^, which suggests a suitable hemocompatibility at concentrations below that value [7]. Thus, the production of CopNc could be an interesting choice for pulmonary administration, since it can prevent the hemolytic potential observed for free Cop.

In addition to the hemolytic potential, the biosafety of Cop and CopNc was evaluated using the MTT assay on alveolar epithelial lung A549 cells. Indeed, in vitro cell culture models are an essential tool for the primary testing of any formulation before proceeding toward ex vivo and in vivo testing. They offer interesting advantages, such as the ability to evaluate toxic effects on specific cell types [47]. Additionally, the MTT assay, a remarkable colorimetric method based on mitochondrial enzymatic activity, is still widely used in the cytotoxicity/biosafety evaluation of different materials, since it is easy to perform, safe, and presents high reproducibility [50]. Moreover, the alveolar epithelial lung A549 cell line, which is derived from human pneumonocytes type-2, is a well-established model for in vitro testing of pulmonary absorption, imuno-oncology, and toxicology [51] This cell line is also suggested by the OECD as a possible model for genotoxicity assays [13].

**Figure 4:**
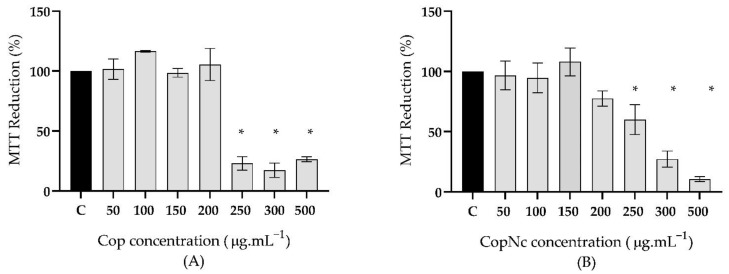
MTT reduction (%) of A549 cells after 24 h of treatment with free copaiba oil (Cop) (**A**) and copaiba oil-loaded nanocapsules (CopNc) (**B**) at concentrations from 50 to 500 µg·mL^−1^ based on the theoretical amount of Cop; *: *p* < 0.05.

The MTT assay on the A549 cell line was performed for Cop and CopNc at concentrations from 50 to 500 μg·mL^−1^ after 24 h of treatment (Figure 4). Cop (Figure 4A) and CopNc (Figure 4B) did not show significant reduction (*p* > 0.05) in the metabolism of MTT by mitochondria enzymes at concentrations of 50, 100, 150, and 200 μg·mL^−1^, suggesting suitable biocompatibility. Nonetheless, at concentrations of 250, 300, and 500 μg·mL^−1^, both Cop and CopNc showed mitochondria enzyme activity reduction above 30% compared to the control (*p* < 0.05), which suggest a cytotoxic potential of the oil and the nanostructured system at these concentrations.

Veiga et al. (2007) also evaluated the cytotoxic effect of copaiba oil at concentrations of 5, 50, and 500 μg·mL^−1^ on peritoneal macrophages using the MTT assay and showed a significant reduction in the mitochondria enzyme activity at concentrations of 500 μg·mL^−1^ [52]. These results highlight how copaiba oil may interact with cells by in vitro assessments, which could lead not only to different toxicological profiles, but also to the safe concentrations that can be used in phytotherapeutic medicines development [23]. Moreover, Nigro et al. (2020) evaluated the cytotoxicity of copaiba oil nanoemulsion on fibroblasts and keratinocytes using the MTT assay and found a concentration-dependent mitochondria enzyme activity reduction effect (above 30%) from 300 μg·mL^−1^ to 10 mg.mL^−1^ in both cell types, which is similar to our results with CopNc [53].

It is important to notice that if CopNc at concentrations of 250, 300, and 500 μg.mL^−1^ did not show hemolytic potential (Figure 3B), at these same concentrations, CopNc induced a cytotoxic effect in the MTT assay (Figure 4B). These results could be attributed to different factors, including (i) the incubation time, which for the MTT assay was 24 h and for the hemolysis assay it was only 1 h; and (ii) the number of exposed cells, which was different for each assay. In addition, the toxicity profile of any substance may be different for specific cell types. Therefore, the results for CopNc could suggest that at the aforementioned concentrations the cytotoxic mechanism of the system was mitochondria enzyme-related; consequently, it could not be observed at the hemolysis assay once matured human erythrocytes do not have mitochondria [54]. Nonetheless, both results highlighted essential information for the toxicological screening of CopNc once the polymeric nanostructured system could come in contact with different microenvironments on further pulmonary administration. Overall, Cop and CopNc showed suitable biocompatibility in both MTT and hemolysis assays at concentrations of 50, 100, 150, and 200 μg·mL^−1^.

In addition to the hemolytic potential and the MTT assay, another important tool to evaluate the safety aspect concerning new molecules or new materials for biomedical applications is their genotoxicity. This approach has often been neglected; however, it is a crucial parameter for technological regulation and risk assessment for several types of materials [18]. For this reason, genotoxicity tests for drug validation are required by regulatory agencies, such as the Food and Drug Administration (FDA) in the USA, the European Medicines Agency (EMA) in the European Union, and the Agência Nacional de Vigilância Sanitária (ANVISA) in Brazil) [12].

Among the available genotoxicity tests, the Cytokinesis-Block Micronucleus (CBMN) assay has become one of the standard cytogenetic assays for genetic toxicology in human and mammalian cells due to its suitable reproducibility and reliability [18]. This assay allows the frequency evaluation of nuclear alterations, such as micronuclei (MN), nucleoplasmatic bridges (NPB), and nuclear buds (NBUD) on binucleated cells exposed to different materials. In addition, the nuclear division index (NDI) can also be assessed, which is a parameter that indicates the ability of a material to interfere with cell proliferation.

Therefore, after the treatment with Cop and CopNc at 200 μg·mL^−1^ (the highest non-cytotoxic concentration), A549 binucleated cells were counted and the frequency of MN, NPB, NBUD, and NDI resulting from DNA damages was analyzed (Table 3). There are many complex molecular mechanisms regarding the formation of MN, NPB, and NBUD; nonetheless, they could be related to chromosome loss, dicentric chromosomes, and gene amplification, respectively [55]. Under the tested conditions, both Cop and CopNc did not induce DNA damage compared to the negative control (*p* > 0.05), which indicates that both Cop and CopNc are also genetically biocompatible. In addition, the frequency of multinucleated cells was counted, and the NDI results showed that these samples also did not affect cell proliferation (*p* > 0.05), which corroborates the MTT assay results (Figure 3).

Similarly, Almeida et al. (2012) evaluated the genotoxic potential of copaiba oil in polychromatic erythrocytes through the CBMN assay and showed that the oil did not induce DNA damage in those cells [56]. In contrast, Cavalcanti et al. (2006) evaluated the genotoxicity of kaurenoic acid isolated from the oil of *C. langsdorffii* at a concentration range from 2.5 to 60 μg·mL^−1^ and found a concentration-dependent increase in MN frequency of hamster lung fibroblasts exposed to the compound; and at 60 μg·mL^−1^, the frequency was similar to the positive control [14]. These results suggest that the Cop complex matrix can interact differently according to the proportion/quantity of the chemical compounds and promote less damage to the cells when compared to its isolated compounds.

Moreover, the genotoxicity of CopNc had not been investigated to this date. Nonetheless, regarding the use of PCL, an ingredient for nanocapsules production, our results are in agreement with Kapustová et al. (2021), who developed PCL nanocapsules with natural oils from *Thymus capitatus* and *Origanum vulgare*, showing that both systems did not induce DNA damage on keratinocyte cells using the comet assay [57]. In addition, Dalcin et al. (2019) developed PCL nanocapsules with the flavonoid dihydromyricetin and showed that the system also did not induce DNA damage [58]. These data suggest that PCL used in the nanocapsules production contributed to the observed genetic biocompatibility.

Overall, it is possible to highlight that both Cop and CopNc did not induce hemolysis in human erythrocytes or cytotoxicity and genotoxicity in A549 cells at concentrations from 50 to 200 μg·mL^−1^, which suggests that both the oil and the system are safe and biocompatible against lung cells at such concentrations. These results represent a step toward the development of nanostructured-based therapies for the pulmonary administration of copaiba oil.

## 4. Conclusions

In the present study, the production and biocompatibility of CopNc aiming for a prospective pulmonary delivery were evaluated to ensure that this system presents suitable physicochemical properties and biosafety against lung cells, before moving toward pharmacological studies. The system was successfully produced and showed particle size distribution of approximately 200 nm and a negative zeta potential, parameters that remained stable for 30 days of analysis. In addition, to evaluate the biocompatibility of CopNc, a toxicological screening approach was designed to assess the ability of both Cop and CopNc to maintain erythrocyte compatibility and lung cells’ integrity on different cell compartments (mitochondria and nucleus). For this purpose, hemolytic potential, MTT, and CBMN assays were carried out. Both Cop and CopNc did not alter cells’ integrity at concentrations up to 200 μg·mL^−1^. Overall, these results highlight the biosafety of both Cop and CopNc and the prospective use of CopNc in future pulmonary administration and phytotherapeutic dosage formulation.

## Figures and Tables

**Table 1:** Chemical characterization of copaiba oil by gas chromatography with a mass spectrometer detector (GC-MS).

Compound	SiCop Area (%)	SiCop RT (min)	Cop Area (%)	Cop RT (min)
(-)-beta-Santalene	-	-	0.2	24.9
(-)-Cyperene	-	-	0.3	23.0
(-)-Isocaryophyllene	-	-	3.6	26.4
(-)-α-Panasinsen	0.1	24.5	-	-
(+)-b-Funebrene	-	-	0.2	24.8
I-β-Famesene	2.1	22.9	-	-
(E)-β-Famesene	-	-	0.9	25.4
(E)-γ-Bisabolene	0.1	24.7	-	-
(Z)-Oleic acid	3.2	38.6	-	-
6,9-Guaiadene	0.4	22.6	-	-
Annonene	0.2	37.0	-	-
Caryophyllene oxide	0.7	26.0	4.8	30.2
cis-(-)-ª,4a,5,ª,9a-Hexahydro-3,5,5,9-tetramethyl(1H)benzoc	-	-	0.3	32.6
cis-α-Bergamotene	-	-	23.1	24.5
cis-α-Bisabolene	2.5	24.0	1.1	27.2
Copaene	0.2	20.8	0.2	22.0
Cyclosativene	0.9	20.6	-	-
Humulene	-	-	0.7	25.2
Humulene epoxide II	-	-	1.2	31.2
Kaur-16-en-18-al, (4α)-	3.3	39.5	-	-
Lanceol, cis	0.1	26.8	-	-
Limona ketone	-	-	0.1	11.9
Linoleic acid	4.8	38.5	-	-
Methyl kolavenate	0.3	41.0	-	-
ni	-	-	0.7	32.9
ni	-	-	0.7	33.5
ni	-	-	0.5	31.5
ni	-	-	0.3	31.7
ni	-	-	0.3	27.6
ni	-	-	0.2	32.2
ni	0.2	28.7	-	-
ni	-	-	0.2	34.3
ni	-	-	0.2	28.2
ni	-	-	0.2	31.4
ni	-	-	0.1	30.8
ni	-	-	0.1	31.0
ni	-	-	0.1	45.6
ni	0.1	27.3	-	-
ni	0.1	30.0	-	-
Sesquiphellandrene	-	-	0.8	28.0
Sesquithujene	-	-	0.4	22.7
trans-α-Bisabolene	3.8	25.0	-	-
α-Bergamotene	22.3	22.4	0.1	23.7
α-Bisabolene	-	-	1.8	28.7
α-Bisabolol	-	-	0.7	34.0
α-Cyprene	0.8	21.5	-	-
α-Selinene	3.6	23.9	0.4	26.8
β-Bisabolene	36.9	24.2	50.2	27.4
β-Caryophyllene	2.9	22.0	5.1	23.8
β-Elemene	3.1	21.3	-	-
β-Selinene	5.2	23.7	-	-
β-Sesquiphellandrene	2.0	24.6	-	-
γ-Muurolene	-	-	0.1	26.1
δ-Elemene	0.1	19.8	-	-

SiCop: copaiba oil after silylation; Cop: copaiba oil; ni: not identified; RT (min): retention time in minutes.

**Table 2:** Stability assessment of copaiba oil nanocapsules stored at room temperature (25 ± 2 °C) over 30 days.

Day	Size (nm) ± SD	PdI ± SD	Zeta Potential (mV) ± SD
1	215 ± 10	0.15 ± 0.01	−18 ± 1
5	216 ± 12	0.14 ± 0.01	−16 ± 3
10	216 ± 4	0.17 ± 0.02	−17 ± 1
15	209 ± 7	0.15 ± 0.03	−21 ± 1
30	219 ± 4	0.17 ± 0.02	−23 ± 2

nm: nanometers; SD: standard deviation; PdI: polydispersity index; mV: millivolts.

**Table 3:** Effect of the exposure of alveolar epithelial lung A549 cells to Cop and CopNc (200 μg·mL^−1^ based on the theoretical amount of Cop) on the frequencies of DNA alterations per 1000 binucleated cells.

Treatment	MN	NPB	NBUD	NDI
NC	8 ± 2.0	6 ± 2	13 ± 2	1.9 ± 0.01
PC	41 ± 3 *	18 ± 4 *	54 ± 6 *	1.8 ± 0.10
Cop	9 ± 2	7 ± 2	17 ± 5	1.9 ± 0.02
CopNc	7 ± 2	4 ± 1	14 ± 4	1.9 ± 0.02

NC: negative control; PC: positive control; MN: micronucleus; NPB: nucleoplasmatic bridges; NBUD: nuclear buds; NDI: nuclear division index; Cop: free copaiba oil; CopNc: copaiba oil-loaded nanocapsules; *: *p* < 0.05.

## Data Availability

Not available.
